# Brazilian medical students’ perceptions of expert versus non-expert facilitators in a (non) problem-based learning environment

**DOI:** 10.3402/meo.v20.26893

**Published:** 2015-04-15

**Authors:** Lucélio B. Couto, Reinaldo B. Bestetti, Carolina B. A. Restini, Milton Faria-Jr, Gustavo S. Romão

**Affiliations:** UNAERP – Medicine School, University of Ribeirão Preto, Ribeirão Preto, Brazil

**Keywords:** problem-based learning, tutorial session, subject-matter expertise, knowledge, education, medical, undergraduate, facilitator behavior

## Abstract

**Background:**

In problem-based learning (PBL), the facilitator plays an important role in guiding the student learning process. However, although content expertise is generally regarded as a useful but non-essential prerequisite for effective PBL facilitation, the perceived importance of content knowledge may be subject to cultural, contextual, and/or experiential influences.

**Aim:**

We sought to examine medical students’ perceptions of subject-matter expertise among PBL facilitators in a region of the world (Brazil) where such active learning pedagogies are not widely used in university or pre-university settings.

**Results:**

Of the 252 Brazilian medical students surveyed, significantly (*p*≤0.001) greater proportions viewed content expert facilitators to be more effective than their non-expert counterparts at building knowledge (95% vs. 6%), guiding the learning process (93% vs. 7%), achieving cognitive learning (92% vs. 18%), generating learning goals (87% vs. 15%), and motivating self-study (80% vs. 15%).

**Discussion/conclusion:**

According to Brazilian medical students, subject-matter expertise among PBL facilitators is essential to the learning process. We believe this widespread perception is due, in large part, to the relative lack of prior educational exposure to such pedagogies.

Problem-based learning (PBL) is a well-recognized teaching approach characterized, among other things, by the student centeredness of the learning process. In this sense, the teacher facilitates the active construction of knowledge rather than merely conveying or purveying it vis-à-vis a lecture, for example (1). The starting point for student learning is typically a problem-solving session (e.g., an actual patient case or theoretical case) that occurs in a small-group setting. The acquired medical knowledge, then, results primarily from the purposeful knowledge acquisition of repeated problem solving.

A small number of students (usually no more than 10) and one professor, referred to as the facilitator,^1^ take part in this tutorial session – whereby a problem is presented to students for discussion. The objective is to develop initial pathways to solving the problem. Because students’ knowledge is incomplete, self-study learning goals are established, followed by a reporting phase where the acquired knowledge is shared and critically reviewed by peers under facilitators’ guidance (2). Because information is not passively conveyed to students by instructors (3), the number of lectures is minimized and much time is spent on developing learners’ self-study skills.

Along with prior student knowledge and the quality of problems discussed, the facilitator plays an important role in virtually all aspects of the PBL tutorial sessions – developing the necessary skills to guide and facilitate the student learning process. First, the facilitator should question, probe, suggest, and/or challenge the ideas that emerge during the problem-solving process (4). Second, they should nurture a sense of mutual respect in which students’ opinions are freely shared, clearly communicated, and readily understood (5). In other words, a good facilitator allows students to propose hypotheses even if they are inaccurate (6) – allowing learners to identify and confront misconceptions and, ultimately, generating knowledge (7). Third, to enhance students’ construction of knowledge, facilitators must use language that is easily and widely understood (6). Fourth, the facilitator should stimulate critical thinking, permitting students to critically assess information presented by their peers (8). Last, facilitators should provide continual, constructive feedback to students on the learning progress (9).

In essence, the facilitator guides a small group of students in a tutorial session on how to solve a problem. To do so, they reinforce the activation of related prior knowledge, the acquisition of new knowledge, the establishment of learning goals for self-study, and the sharing of knowledge gleaned via self-study. This process is believed to nurture the development of several cognitive abilities, including recall, knowledge retention, and problem-solving skills (10).

A relevant and still controversial aspect of this approach is the breadth and depth of content-specific knowledge needed by the facilitator to effectively guide the learning process. There are conflicting data regarding the necessity of subject-matter expertise versus non-subject-matter facilitators. Although some authors have found the former to be superior in instilling cognitive acquisition among learners (5, 6, 11–13), others have not (14–20). Still others have yielded mixed results (21–23), with students’ evaluations of facilitators revealing both subject-matter and process expertise to be necessary for effective learning (24).

Student opinion, however, can be influenced by cultural (24, 25) and contextual characteristics (26). In view of the potential learning implications, it is worthwhile to explore Brazilian students’ perceptions of the facilitator role with regard to subject-matter expertise – because learning in our region is characterized by marked educational deficiencies, a lack of prior PBL exposure in primary education, and limited stimulation in learner-centered approaches (25). Accordingly, this study seeks to examine how Brazilian medical students, as relative PBL ‘novices’, view the effectiveness of expert and non-expert facilitators as a determinant of learning.

## Methods

### Setting

The University of Ribeirão Preto Medical School (UNAERP) began a PBL curriculum in 2003 using non-subject-matter experts as facilitators. These ‘lay’ preceptors, formally schooled in the PBL process, were replaced by content-specific experts in 2013 on the recommendation of our institutional Planning Group. As a result, students in the fifth to eighth stages of medical school^2^ experienced the PBL learning process under the guidance of non-subject-matter facilitators, and could compare it with that of the subject-matter experts. Subject-matter expertise has been previously defined (12), but is essentially background knowledge of the specific subject under discussion; a non-subject-matter facilitator, in contrast, has no training or experience related to the subject at hand. For example, in the cardiovascular disease unit, practicing cardiologists constitute subject-matter experts who are also trained in guiding the PBL learning process. A pediatrician, with identical pedagogical training, would be considered a non-subject-matter expert in this context.

Details of our PBL curriculum have been described elsewhere (25). Briefly, from the first to the fourth stage, students are guided by subject-matter experts in problem solving related to morpho-physiological aspects of medicine, and not to human diseases. The integrated tutorial sessions parallel learning activities in clinical skills and primary care. Typically, in this period of the medical course, subject-matter expert facilitators are basic scientists rather than clinicians. The basic PBL structure involves a tutorial session composed of no more than 10 students, and a subject-matter expert to guide (or facilitate) the learning process. During a specific topic, students participate in two different groups led by two different subject-matter experts. So, two subject-matter experts guide 20 students in the tutorial sessions for a given unit (e.g., cardiovascular disease); in the next unit (there are three units per semester) the same dynamic is repeated. Because the facilitators possess some topical knowledge, students will have three different subject-matter expert facilitators each semester. From the fifth to eighth stage, all subject-matter expert facilitators are physicians specializing in the topic at-hand. The problem-solving tutorial sessions consist of the most common diseases affecting humans. As mentioned above, this activity is also integrated with clinical skills (seeing patients on wards or at ambulatory setting) and primary care.

Subject-matter and non-subject-matter experts have been equally versed in the PBL method prior to facilitating the tutorial sessions – completing more than 20 hours of training consisting of lectures, guided observation, and ‘hands-on’ practice with actual students. This training regimen existed for more than 10 years, giving all facilitators a working knowledge of PBL.

### Student participation

Written informed consent was obtained from students in the fifth to eighth stages of medical training. Prior to the end-of-unit test, participating students anonymously evaluated the roles of facilitators via six key areas as applied to both subject-matter expert facilitator and non-subject-matter experts: 1) knowledge construction; 2) effectiveness to guide the learning process; 3) facilitation of generation of learning goals for self-study; 4) motivating for self-study during the tutorial session; 5) cognitive learning achievement; and 6) preparing the end-of-unit test. Using a 5-point, Likert-type scale ranging from ‘strongly disagree’ to ‘strongly agree’, items were phrased per the following: ‘Knowledge during the tutorial process with the non-subject-matter expert facilitator was very good’. The local Ethical Committee approved the study.

### Statistical analysis

The chi-square test statistic was used to compare students’ ordinal ratings of subject-matter and non-subject-matter expert facilitators, as previously described by Azer et al. (27). Differences of *p*≤0.05 were considered statistically significant.

## Results

All 254 students in the fifth to eighth years of medical training agreed to participate in the study. Of these, two responses were incomplete, and excluded from the study. Of the remaining 252, the average age was 23.4 years of age.

The overwhelming majority (95%) of students, or 239/252, agreed or strongly agreed that the subject-matter expert facilitator was ‘very good’ at building knowledge, whereas only 16 students (6%) agreed similarly for non-subject-matter experts (*p*≤0.001). These same results held true for the remainder of the evaluation items: 1) guiding the learning process (93% vs. 7%); 2) generating self-study learning goals (87% vs. 15%); 3) motivating self-study (80% vs. 15%); 4) achieving cognitive learning (92% vs. 18%); and 5) preparing the end-of-unit test (76% vs. 6%).

## Discussion

These findings clearly show that Brazilian medical students’ believe subject-matter expertise to be a crucial factor in virtually all aspects of the PBL learning process. Because the PBL pedagogy is not provided in our regional pre-university settings (25), only cursory exposure appears to instill in students their own conceptions about the role of content-specific knowledge in facilitating their learning of medical knowledge.

Compared to a non-subject-matter expert, the role of subject experts in facilitating the learning process remains debated in the PBL literature (5, 6, 11–20). Such inconsistencies can be ascribed to several factors – including sample size, sample characteristics, operational definitions of expert and non-expert facilitators, institutional culture, curricular structure, learning outcomes, and previous experience with the PBL method, to list but a few.

Underlying students’ facilitator evaluations are a complex process related to various parameters. Dolmans et al. (28) suggest that facilitator performance rarely shows stability because, among other things, it is related to problem quality, students’ knowledge levels, and the larger group dynamic. They also showed that the same facilitators obtained lower ratings from tutorial groups with low productivity, compared with higher scores from more productive groups (29). Unproductive groups, it appears, need to be stimulated and challenged by a skilled facilitator. In this regard, Schmidt et al. (30) showed that subject-matter expert facilitators utilize their subject-matter expertise toward this end, whereas non-subject-matter expert facilitators utilize their process-facilitation expertise.

In our study, students considered subject-matter expert facilitators’ performance better than that of non-subject-matter experts for their knowledge construction. This is in accordance with findings by Moust et al. (31), who found that law students led by subject-matter expert facilitators scored higher on a cognitive skills test relative to those led by peer facilitators. The authors hypothesized that by virtue of their more elaborate knowledge structures of the topic-at-hand, subject-matter experts are more effective at guiding the restructuring of each student's cognitive skills. However, despite findings by Davis et al. (11) suggesting that students often work independently – and not in response to teacher direction – students’ ratings of the learning process (as well as actual academic achievement) were higher in the subject-matter expert facilitator group than in the non-subject-matter expert facilitator group. Our study findings appear to largely concur.

Conversely, however, McLean (9) found that facilitators emphasized the difficultly in not providing subject-matter expertise to students during the tutorial session – which does not seem to have occurred in our investigation. Furthermore, subject-matter expert facilitators can more easily show how clinical reasoning applies to the problem during the discussion, thus increasing motivation for self-study and knowledge construction (13). It is tempting to speculate that subject-matter experts may have led students to learn through their ability to ask questions that prompted students to consider things they were not able to posit themselves (32).

Another interesting finding is that students considered relevant the subject-matter experts facilitators’ performance better for guiding the learning process. This contrasts with the work by Silver and Wilkerson (33), who observed that subject-matter expert facilitators actually impeded the learning of first-year medical students by providing direct answers to students’ questions, contributing less to the facilitative statements, promoting less exchange, and dominating the discussion process. Level of student may have been one factor in this discrepancy; on the other hand, our findings generally coincide with those of Schmidt (12), who found facilitators’ subject-matter expertise to be an important correlate of academic achievement (as measured on end-of-unit tests) – even among first-year students.

Our investigation similarly shows that the perceived positive effects of a subject-matter expert facilitator can also occur among more advanced medical students. It is conceivable that the documented interplay among subject-matter expert facilitators, the facilitator's commitment to student learning, and the facilitator's ability to communicate in the students’ language, have all been established as relevant in the context of PBL (6).

Our sample of Brazilian medical students also viewed the subject-matter expert facilitator better than non-subject-matter experts at performing to guide students for generating learning goals. Eagle et al. (34) studied the performance of 70 undergraduate medical students in simulated-patient cases, and found that students led by a subject-matter expert facilitator generated twice the number of learning goals – and spent twice the time engaged in self-study – than those led by non-subject-matter facilitators. Because our student sample had no prior undergraduate university background, it is possible that subject-matter experts better recognize and communicate knowledge gaps – and subsequently stimulate the generation of learning goals for self-study.

According to our results, learners’ motivation for self-study was deemed to be enhanced by group facilitation from subject-matter experts. Schmidt et al. (30) analyzed students’ academic performance across 4 years, comparing the impact of expert versus non-expert facilitators. They observed that students led by subject-matter expert facilitators not only achieved more academically, but also dedicated more time to self-study (though these differences varied by year). Our results tend to agree, and it is conceivable that subject-matter experts simply use their backgrounds to better motivate students.

Another finding of this investigation was that students considered relevant quality of the performance of the subject-matter expert facilitators for driving cognitive learning achievement. Steinert (26) studied the responses of 46 first- and second-year medical students concerning the effectiveness of facilitators at guiding student acquisition of metacognitive skills. She observed that the knowledge of and capacity to highlight the clinical importance of cases, together with the ability to expand their clinical relevance, were important facilitation skills. Hay and Katsikitis (35), in their study of 144 fourth-year medical students, showed that learners led by non-subject-matter experts achieved higher scores for task management skills, but students guided by subject-matter expert facilitators achieved higher examination scores.

Although our results do not dispute this, one possibility is that subject-matter expert facilitators interact more productively with students and/or have a greater interest in student development, thus facilitating knowledge/skills acquisition (6).

Finally, Brazilian medical students believed that subject-matter expert facilitators prepared the end-of-unit tests more sufficiently than their non-subject-matter counterparts. This is of obvious importance, because scores on the end-of-unit test determines whether they pass or fail the unit. It is conceivable that greater topical knowledge and a clearer understanding of unit objectives allowed subject-matter experts to formulate higher-level questions for students’ development and to assess their achieved knowledge.

These study findings are not without potential limitations. First, we compared students’ perceptions retrospectively – which may be subject to some recall bias. Second, we did not measure student achievement objectively – relying instead on learners’ subjective assessments. Third, we did not assess students’ opinions on the role of the subject-matter expert facilitator in the first year of medical school, so our findings may not generalize to all learners. Finally, our tool for measuring the expertise can be overly basic, and likely did not allow us to reach some variability that exists among facilitators’ knowledge bases.

## Conclusion

With the aforementioned shortcomings aside, the importance of subject-matter experts in facilitating the learning process in a PBL environment are seen by Brazilian medical students as crucially important to the learning process. Although we cannot offer direct empirical evidence, we believe that a lack of prior cultural exposure to such active-learning pedagogies plays a role in this assessment.

## Footnotes

1 Facilitator, as used here, is synonymous with tutor or preceptor.

## Footnotes

2 Stage represents one of 12 semesters of the medical curriculum when students attend different learning unities.
